# Supplementary material for: Impact of recent climate extremes on mosquito-borne disease transmission in Kenya
Source: PLoS Negl Trop Dis. 2021 Mar 18;15(3):e0009182. doi: 10.1371/journal.pntd.0009182 (PMC7971569; doi:10.1371/journal.pntd.0009182)
Supplement: S1 Appendix — (DOCX) [file pntd.0009182.s001.docx]

Due to a lack of historical data for ambient air temperatures, the study makes use of satellite-derived land surface temperatures (LST) instead. The difference between ambient air temperature and LST causes an exchange of energy at the planetary boundary level. LST represents the combined temperature of all earth surface elements, including vegetation, soil, etc. Compared to surface air temperatures, LST better represents vector habitat conditions; as a result, variations in LST influence mosquito habitat suitability under varying climatic conditions. Moreover, it is important to note that there is a strong relationship between LST and ambient air temperature (Fig 2). However, while there is a strong linear relationship between these two variables, LST is not a perfect proxy for ambient air temperature. On average, LST values on any given day are 4.4°C greater compared to ambient air temperature, with differences ranging from -4.3°C to +15.3°C due to turbulence near the surface (S6 Fig).

LST values are derived from daytime and nighttime thermal infrared measurements using the validated LST algorithm described by Wan et al. Cloud screening is performed using the MODIS cloud mask product prior to calculation of LST values [1]. The estimated accuracy of LST values is ± 1 °C.

**References**

1. Wan, Z., Zhang, Y., Zhang, Q. & Li, Z. Validation of the land-surface temperature products retrieved from Terra Moderate Resolution Imaging Spectroradiometer data. Remote Sens. Environ. 2002;83, 163–180.
