# Supplementary material for: Impact of recent climate extremes on mosquito-borne disease transmission in Kenya
Source: PLoS Negl Trop Dis. 2021 Mar 18;15(3):e0009182. doi: 10.1371/journal.pntd.0009182 (PMC7971569; doi:10.1371/journal.pntd.0009182)
Supplement: S2 Appendix — (DOCX) [file pntd.0009182.s002.docx]

*Ovitrap*: An ovitrap is a device that traps laid mosquito eggs on wet paper. Ovitraps are placed in selected households at ground level. Indoor traps are placed in dark corners near cloth racks and at least two meters from water. Outdoor traps are placed around the house, usually near vegetation. For five days every month, ovitraps are exposed for oviposition, as *Ae. aegypti* adults lay eggs. Eggs are then taken to the laboratory and observed and counted using stereomicroscopy. Larval emergence into adults in tap water is necessary for species identification. Egg density is thus used as a marker of abundance of *Aedes aegypti* mosquitoes.

*Pupal trapping*: Pupal trapping involves the sampling of mosquitoes in their post-larval stages, usually found in containers of standing water (buckets, drums, tires, etc.). This is a traditional survey form to monitor mosquito populations and requires verbal consent from homeowners. Such sampling occurs once a month between 7 am and 10 am. Artificial and natural containers in and around households are surveyed by field teams for pupae and larvae and collected using ladles and pipettes. The pupae and larvae are taken to the laboratory for identification.

*Prockopack*: The Prokopack aspirator is a device that allows for direct sampling of resting mosquitoes indoors, on walls, and under the roof. A team of two collectors collect mosquitoes from 11 am to 3 pm in selected homes that provide verbal consent once a month. Collected tins containing mosquitoes are transported to the laboratory for counting and identification.

*BG- trapping*: A CO_2_-baited BG trap is a large funnel that mimics convection currents and gases created by a human body to attract and trap mosquitoes. Traps are placed indoors in houses that provide verbal consent. Traps are placed and powered for 24-hour periods, and then subsequently replaced for two weeks per month. Collected mosquitoes are taken to the laboratory for counting and identification.
