# Supplementary material for: Impact of recent climate extremes on mosquito-borne disease transmission in Kenya
Source: PLoS Negl Trop Dis. 2021 Mar 18;15(3):e0009182. doi: 10.1371/journal.pntd.0009182 (PMC7971569; doi:10.1371/journal.pntd.0009182)
Supplement: S3 Appendix — (DOCX) [file pntd.0009182.s003.docx]

We conducted a Kruskal-Wallis test by ranks, a one-way nonparametric form of an ANOVA based on ranks. The test was used to assess whether the distribution of vector abundance with a one-month lag is significantly different following both extreme rainfall events and extreme temperature events. Extreme rainfall events considered differences among three groups, including periods defined as “flood,” “drought,” and “normal rainfall.” Extreme temperature events considered differences among three groups, including “heat wave,” “cold wave,” and “normal LST.” In order to compare vector abundance following an extreme climate month to that following a “normal” month, we conducted a Wilcoxon rank sum test, a two-sample nonparametric comparison. An identical analysis was done for DENV infection two months following the anomaly, as this lag period is expected based on the cycle of infection.

Additionally, we conducted stratified Kruskal-Wallis tests by ranks in order to assess potential site-specific effects influencing the distribution of vector abundance one month following climate anomalies. Additional bivariate analyses were stratified using several methods: 1) All Study Sites, 2) Rural (Chulaimbo and Msambweni) vs Urban (Kisumu and Ukunda), 3) Western (Kisumu and Chulaimbo) vs Coastal (Msambweni and Ukunda), 4) Individual Study Sites. Unique thresholds were established for each method of stratification based on the lower and upper 10% of anomalies.
