# Supplementary figures and images for: Impact of recent climate extremes on mosquito-borne disease transmission in Kenya
Source: PLoS Negl Trop Dis. 2021 Mar 18;15(3):e0009182. doi: 10.1371/journal.pntd.0009182 (PMC7971569; doi:10.1371/journal.pntd.0009182)

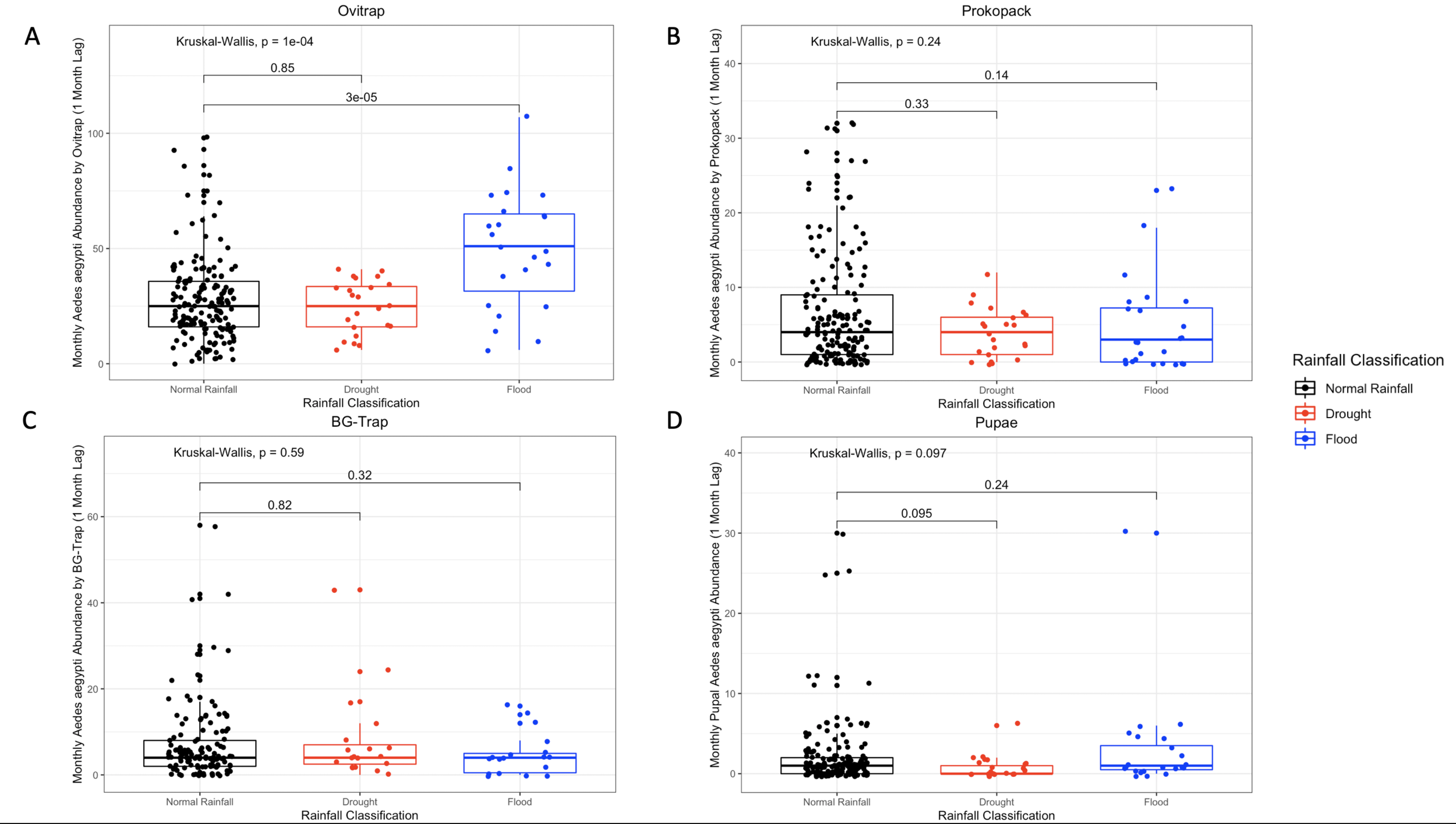

Supplement: S1 Fig — Boxplot of vector abundance by A) ovitrap B) Prokopack C) BG-trap and D) pupal trap one month following anomaly classified as normal, drought, or flood. Wilcoxon test p-values displayed between groups. (TIF) [file pntd.0009182.s004.tif]

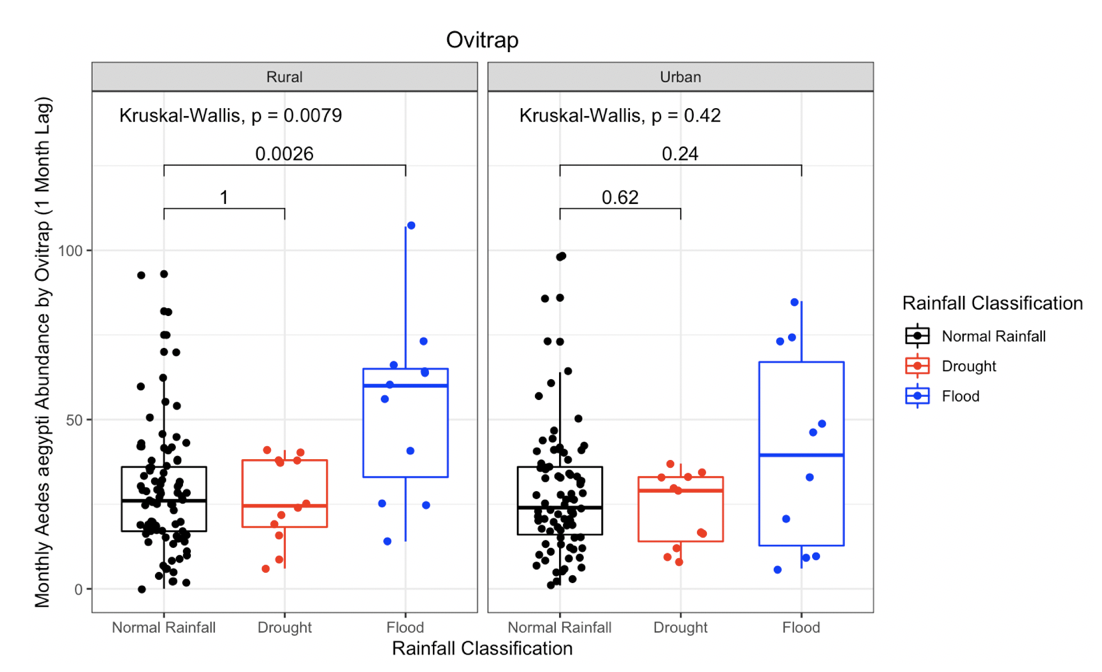

Supplement: S2 Fig — Boxplot of Ae. aegypti egg abundance one month following anomaly classified as normal, drought or flood. Stratified by rural (Chulaimbo and Msambweni) and urban (Kisumu and Ukunda) sites. Wilcoxon test p-values displayed between groups. (TIF) [file pntd.0009182.s005.tif]

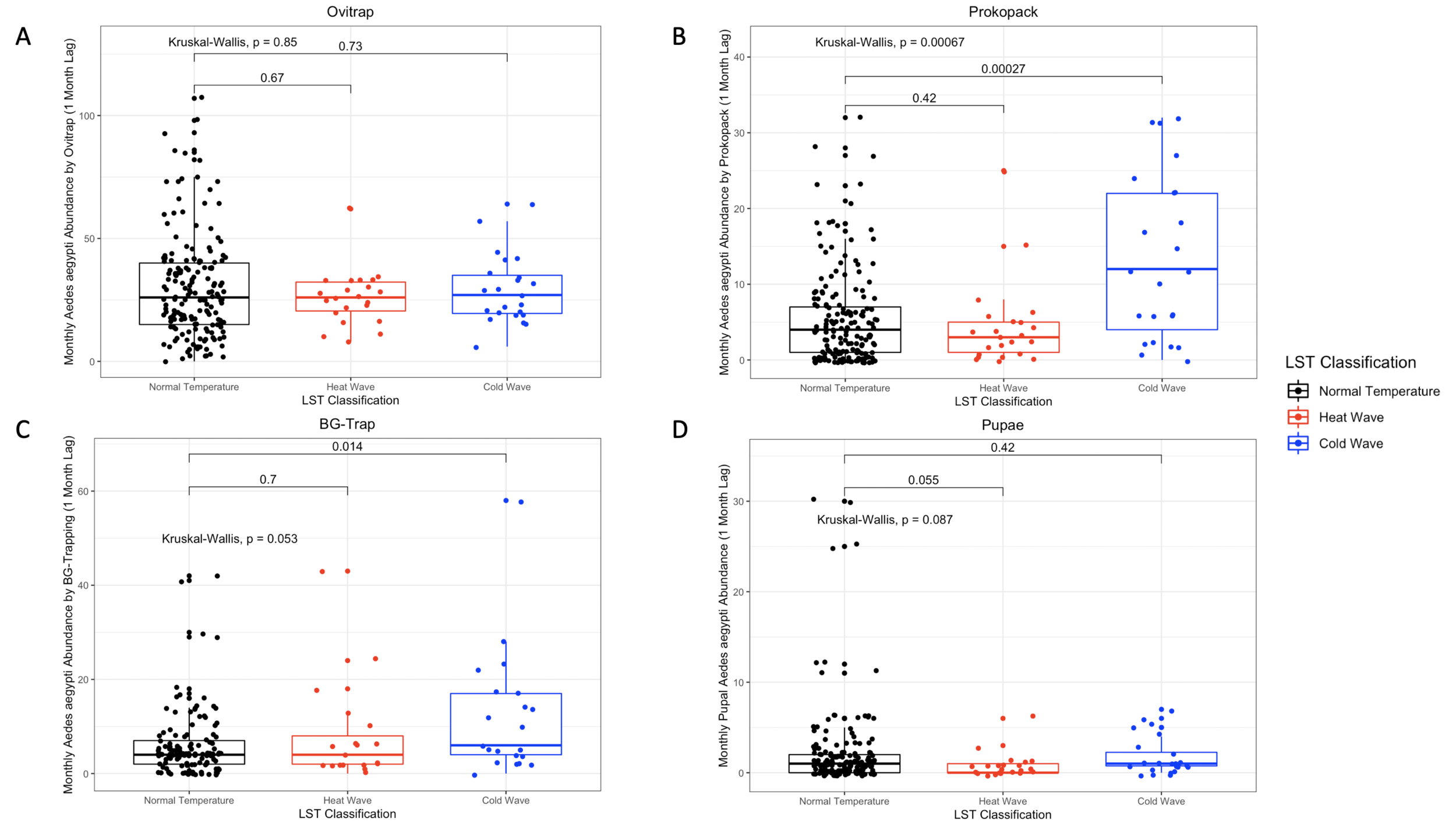

Supplement: S3 Fig — Boxplot of vector abundance by A) ovitrap B) Prokopack C) BG-trap and D) pupal trap one month following anomaly classified as normal, heat wave, or cold wave. Wilcoxon test p-values displayed between groups. (TIF) [file pntd.0009182.s006.tif]

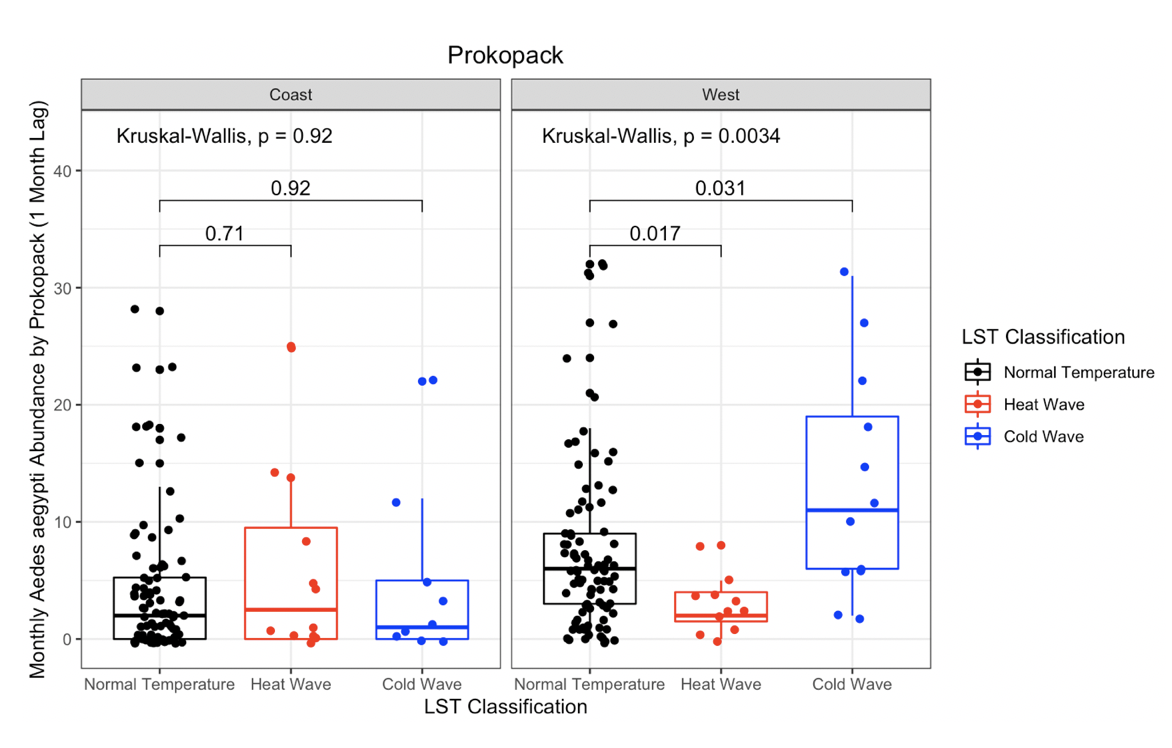

Supplement: S4 Fig — Boxplot of adult Ae. aegypti abundance (Prokopack) one month following anomaly classified as normal, heat wave or cold wave. Stratified by western (Kisumu and Chulaimbo) and coastal (Ukunda and Msambweni) sites. Wilcoxon test p-values displayed between groups. (TIF) [file pntd.0009182.s007.tif]

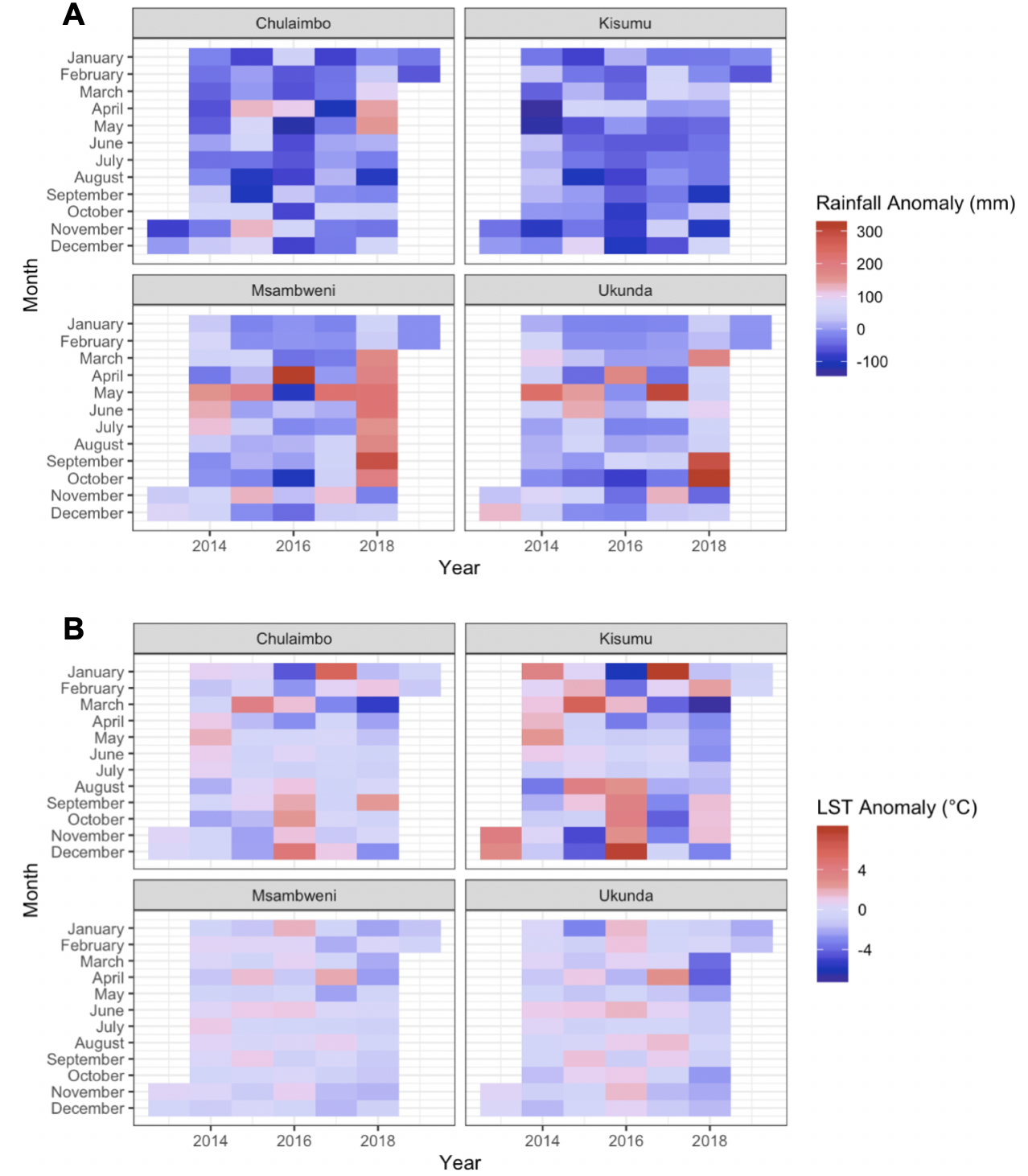

Supplement: S5 Fig — Heat map displaying monthly anomaly severity for A) accumulated rainfall and B) average LST between November 2013—February 2019, stratified by study sites. (TIF) [file pntd.0009182.s008.tif]

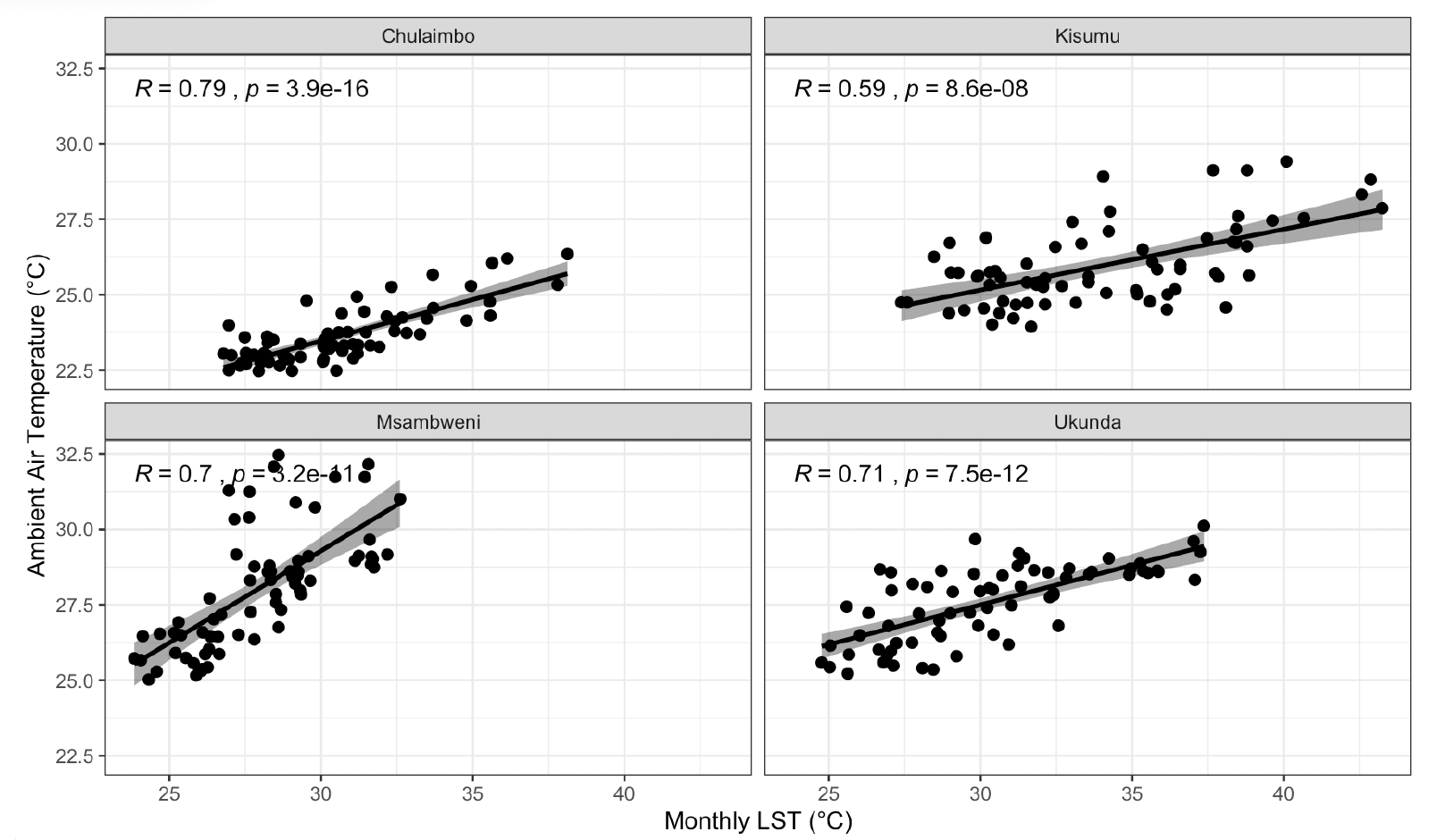

Supplement: S6 Fig — Across all four study sites, there is a strong correlation (R > 0.50, p < 0.05) between land surface temperature and ambient air temperatures between November 2013 –February 2019; however, there is clear variability between the two measurements. (TIF) [file pntd.0009182.s009.tif]
