# Supplementary material for: Impact of recent climate extremes on mosquito-borne disease transmission in Kenya
Source: PLoS Negl Trop Dis. 2021 Mar 18;15(3):e0009182. doi: 10.1371/journal.pntd.0009182 (PMC7971569; doi:10.1371/journal.pntd.0009182)
Supplement: S1 Table — Mean and standard deviation of monthly LST (°C), LST anomaly (°C), monthly rainfall (mm), rainfall anomaly (mm), monthly ambient air temperature (°C), and monthly humidity (%) between previously defined groups of “flood,” “drought,” and “normal rainfall.” LST and rainfall anomalies refer to difference between observed monthly values and long-term means. p-values indicate significance values from Kruskal-Wallis Rank Sum tests among three groups. *p ≤ 0.05, ** p ≤ 0.01, *** p ≤ 0.001 for Wilcoxon-test where “Normal Rainfall” is considered the reference group. (DOCX) [file pntd.0009182.s010.docx]

| **Variable** | **Flood (N = 26)** | **Drought (N = 26)** | **Normal Rainfall (N = 204)** | **p-Value** |
| --- | --- | --- | --- | --- |
| **Monthly LST (°C)**,  Mean (SD) | 27.40 (1.93)*** | 33.60 (4.00)*** | 30.91 (3.67) | **<0.001** |
| **LST Anomaly (°C)**,  Mean (SD) | -1.32 (1.05)* | 1.76 (2.01)*** | -0.43 (1.87) | **<0.001** |
| **Monthly Rainfall (mm)**, Mean (SD) | 315.91 (97.72)*** | 35.20 (32.49)*** | 92.33 (65.54) | **<0.001** |
| **Rainfall Anomaly (mm)**, Mean (SD) | 194.28 (62.11)*** | -90.61 (17.30)*** | 8.85 (44.26) | **<0.001** |
| **Monthly Ambient Air Temperature (°C)**,  Mean (SD) | 26.98 (1.91) | 25.17 (1.62)** | 26.48 (2.32) | **0.008** |
| **Monthly Humidity**, Mean (SD) | 80.60 (3.38)*** | 59.15 (12.09)*** | 67.60 (12.08) | **<0.001** |
