# Supplementary material for: Impact of recent climate extremes on mosquito-borne disease transmission in Kenya
Source: PLoS Negl Trop Dis. 2021 Mar 18;15(3):e0009182. doi: 10.1371/journal.pntd.0009182 (PMC7971569; doi:10.1371/journal.pntd.0009182)
Supplement: S2 Table — Mean and standard deviation of monthly LST (°C), LST anomaly (°C), monthly rainfall (mm), rainfall anomaly (mm), monthly ambient air temperature (°C), and monthly humidity (%) between previously defined groups of “heat wave,” “cold wave,” and “normal LST.” LST and rainfall anomalies refer to difference between observed monthly values and long-term means. p-values indicate significance values from Kruskal-Wallis Rank Sum tests among three groups. Note: *p ≤ 0.05, ** p ≤ 0.01, *** p ≤ 0.001 for Wilcoxon-test where “Normal LST” is considered the reference group. (DOCX) [file pntd.0009182.s011.docx]

| **Variable** | **Heat Wave (N = 26)** | **Cold Wave (N = 26)** | **Normal LST**  **(N = 204)** | **p-Value** |
| --- | --- | --- | --- | --- |
| **Monthly LST (°C)**,  Mean (SD) | 36.52 (4.01)*** | 29.99 (2.05) | 30.21 (3.35) | **<0.001** |
| **LST Anomaly (°C)**,  Mean (SD) | 3.39 (1.78)*** | -3.71 (1.11)*** | -0.33 (1.02) | **<0.001** |
| **Monthly Rainfall (mm)**, Mean (SD) | 66.75 (46.17)* | 161.19 (97.55) | 108.02 (100.24) | **0.001** |
| **Rainfall Anomaly (mm)**, Mean (SD) | -45.53 (44.66)*** | 43.22 (85.64) | 22.36 (79.24) | **<0.001** |
| **Monthly Ambient Air Temperature (°C)**,  Mean (SD) | 26.45 (1.86) | 25.78 (1.75)) | 26.47 (2.36) | 0.338 |
| **Monthly Humidity**, Mean (SD) | 55.65 (13.58)*** | 62.18 (12.03)*** | 70.39 (11.19) | **<0.001** |
