# Supplementary material for: Impact of recent climate extremes on mosquito-borne disease transmission in Kenya
Source: PLoS Negl Trop Dis. 2021 Mar 18;15(3):e0009182. doi: 10.1371/journal.pntd.0009182 (PMC7971569; doi:10.1371/journal.pntd.0009182)
Supplement: S3 Table — (DOCX) [file pntd.0009182.s012.docx]

| **Variable** | **Low** | **Intermediate** | **High** |
| --- | --- | --- | --- |
| **Ovitrap *Ae. aegypti* abundance**, *N (%)* | 0-15 (0-22%) | 16-40 (22-78%) | 41-107(78-100%) |
| **Prokopack *Ae. aegypti* abundance**, *N (%)* | 0 (0-20%) | 1-9 (20-80%) | 10-32(80-100%) |
| **BG-Trap *Ae. aegypti* abundance**, *N (%)* | 0-1 (0-17%) | 2-12 (17-83%) | 13-58 (84-100%) |
| **Pupal Trapping *Ae. aegypti* abundance**, *N (%)* | 0 (0-38%) | 1-4 (39-89%) | 5-30 (90-100%) |
| **Confirmed dengue infections**, *N (%)* | 0-6 (0-90%) | NA | 7-23 (90-100%) |
