# Supplementary material for: Impact of recent climate extremes on mosquito-borne disease transmission in Kenya
Source: PLoS Negl Trop Dis. 2021 Mar 18;15(3):e0009182. doi: 10.1371/journal.pntd.0009182 (PMC7971569; doi:10.1371/journal.pntd.0009182)
Supplement: S4 Table — Missing data was excluded from the regression models. (DOCX) [file pntd.0009182.s013.docx]

| **Variable** | **Number of Missing Observations** |
| --- | --- |
| **Ovitrap *Ae. aegypti* abundance** | 41 |
| **Prokopack *Ae. aegypti* abundance** | 40 |
| **BG-Trap *Ae. aegypti* abundance** | 91 |
| **Pupal Trapping *Ae. aegypti* abundance** | 35 |
| **Confirmed dengue infections** | 17 |
