# Supplementary material for: Impact of recent climate extremes on mosquito-borne disease transmission in Kenya
Source: PLoS Negl Trop Dis. 2021 Mar 18;15(3):e0009182. doi: 10.1371/journal.pntd.0009182 (PMC7971569; doi:10.1371/journal.pntd.0009182)
Supplement: Attachment — Submitted filename: PNTD-D-20-01178.docx [file pntd.0009182.s014.docx]

The Editor

PLoS NTD

**RE:** PNTD-D-20-01178

**Title: Impact of Recent Climate Extremes on Mosquito-Borne Disease Transmission in**

**Kenya**

The paper presents results of the effect of land surface temperature, rainfall and season on mosquito vectors of dengue fever virus and infection patterns in children in four sites in Kenya – Ukunda, Masambweni, Chulaimbo and Kisumu. Data were collected between 2013 and 2019 and temperature and rainfall anomalies derived for the periods 2000 – 2019 and 1983 to 2019, respectively, and used as predictors. Statistically significant results were obtained for mosquito populations but not with infection rates.

Major comments

The paper however has a number of weaknesses that renders it unsuitable for this journal.

1. The authors have used satellite climate (LST and rainfall) data with no comparison, reference to ground measurements from meteorological stations in the study areas. Satellite data have lower resolution and always averaged over vast regions. It often makes a lot of sense to use such data for analyses conducted over wide geographical areas and not for areas that are too close to present meaningful comparisons. In fact anomalies for rainfall data are estimated at the county level and assigned to study sites. This effectively reduces the power of the study and it might have contributed to the lack of effect of predictors on infection patterns in children.

2. No data are provided to show the ages of children used, their distribution by households etc to gauge whether their infection patterns provided a good representation of the study areas used. If young children were used, then it can be argued that their force of infection was too low to be of practical significance given that they are raised in doors most of the time. Published data show that Aedes mosquitoes are day biters. They are more likely to transmit the disease during the day than the night like other mosquitoes such as Anopheles which transmits malaria. Older people are expected to come into contact with mosquitoes as they work outside.

Minor comments

Line 163 – 165: Reference periods for the estimation for anomalies for LST and rainfall differ. This is confusing, it might be better to use a common reference time period for both.

Line 177 – Flood is categorised as a positive rainfall deviation above 90% threshold. His categorization is done without considering the topography of the study areas not the length of time this this anomaly has been observed. A positive anomaly for one month is not enough to cause floods in some places. The interpretation of the effect of this variable therefore is not justifiable. This is why in Line 291, it is observed that flooding increases egg load. This is not usually true especially if flushing was to occur

Line 185: Drought is defined as a dip in rainfall levels by at least 10%. However, drought usually occurs where there has been prolonged period of dry weather for several months. This classification, as that identified above, brings confusion and makes data interpretation difficult to follow

Line 216 Replace “is” with “was”

Line 225 Indicate whether the ordinal logistic regression model used satisfied the proportional odds assumption

Line 227 Mosquito counts are not expected to follow a normal distribution. Many studies often transform the raw data e.g. using log transformation

Line 245 what is the justification for using 7 as the cut off for the number of cases

Line 257 What are the groups being considered?

Line 274 - 276 fits well in the methodology. It is trying to justify the type of analysis done

Line 376 Urbanization not introduced in the methodology

Line 392 How can heat wave affect Chulaimbo alone, and not together with the other site in the same county? Are there other variables that were not captured
