# Supplementary material for: Impact of recent climate extremes on mosquito-borne disease transmission in Kenya
Source: PLoS Negl Trop Dis. 2021 Mar 18;15(3):e0009182. doi: 10.1371/journal.pntd.0009182 (PMC7971569; doi:10.1371/journal.pntd.0009182)
Supplement: Attachment — Submitted filename: Reviewers Responses to Questions.docx [file pntd.0009182.s015.docx]

Reviewer's Responses to Questions

Key Review Criteria Required for Acceptance?

As you describe the new analyses required for acceptance, please consider the following:

Methods

-Are the objectives of the study clearly articulated with a clear testable hypothesis stated?

-Is the study design appropriate to address the stated objectives?

-Is the population clearly described and appropriate for the hypothesis being tested?

-Is the sample size sufficient to ensure adequate power to address the hypothesis being tested?

-Were correct statistical analysis used to support conclusions?

-Are there concerns about ethical or regulatory requirements being met?

1. Reviewer #1: No

**Response: Objectives of the study are articulated in lines 153-155 and the study design/methods are described individually for the climate, vector, and dengue data in lines 180-284. The Kenyan study sites have been clarified in the text (lines 180-182) and include Ukunda, Kisumu, Chulaimbo, and Msambweni. We used several different statistical models to understand how the magnitude and categorization of anomalies affect dengue transmission (lines 186-347). Ethical requirements are acknowledged in lines 170-177.**

2. Reviewer #2: The paper is addressing an important component of climate change in relation to an arbovirus infections. The effects of climate extremes on vector-born diseases is not well understood. Attempts have been made to use statistical, dynamic and process based model to explain the interaction of the various meteorological variables and their final impact on vectors and pathogens. The complexity of a model does not0 allways provide the expected results. A clear understanding of the biology of transmission in relationship to meteorological variables is critical in modelling transmission. In most cases the relationship between temperature and the viral extrinsic incubation period is often non-linear. Lags of unknown periods are also required for the breeding habitats to stabilize. Some extreme rainfall and temperature are lethal vectors and the virus. In other cases rainfall is the only controlling factor as the temperature conditions are close to ideal for hypertransmission.The strength of this paper lies in its exploratory aproach in finding the relationship between extreme climate events and dengue transmission. However it suffers from the use of satellite derived data. Among the shortfall of this data is failure to disentangle the impacts of variability in the minimum and maximum temperature. In some sites over 95% of the temperature variability comes from the maximum temperature while in some sites the minimum temperature may contribute as much as 20% of the anomalies. Thus while the mean temperature may fail to reveal the full extent of anomalies, the max and min temperatures could be telling a different story between sites.

**Response: We appreciate the Reviewer highlighting the value of this work. Disentangling the contribution of minimum and maximum temperature in driving anomalies is a very interesting and important point to consider. We now address this in the Discussion, explaining that minimum and maximum LST data are currently unavailable, but future studies should consider investigating this issue with in situ measurements where available.**

**Lines 910-913: “Minimum and maximum temperature values can impact temperature variability to differing extents, so future studies should consider temperature anomalies in the context of these extreme values rather than the mean. We are unable at the moment to reconstruct the data as produced and provided to tease out the effect of minimum and maximum temperatures. This can be considered in future studies.”**

3. Temperature and rainfall threasholds define the windows of transmission and it is importsnt to identify these threasholds while developinng statistical models. Care must be taken in usind statistically defined extreme meteorological conditions without making reference to the trsnsmission windows.

**Response: Our categorized anomalies have been defined as “monthly” anomalies. Additionally, we have clarified them as monthly anomalies more often throughout the text so that the time window is clear. Additionally, we have acknowledged the importance of such transmission windows and thresholds in the discussion.**

**Lines 928-929: “Furthermore, it is necessary to consider “anomalies” in the context of established biologically relevant thresholds for both temperature and rainfall.”**

4. Reviewer #3: The objectives of the study, identify how periods of extreme rainfall and temperature affect mosquito abundance and the risk of dengue infection, is clearly articulated, but not formulated in the form of a hypothesis. The study design appropriate to address the stated objectives, the data set is clearly described.

Monthly mean land surface temperatures was gridded at 0.05° while rainfall at 0.1°. Do you expect any scale dependency in the results comparing temperature with rainfall effects? The measurement of the vector abundance using different types of traps is well thought out.

**Response: While the nominal resolution of the LST is 0.05° and Rainfall is 0.1°, we extracted data for a 30 x 30 km centered on each study location so the data were comparable. This removed or minimized the scale dependency in the comparisons. This has been done for previous studies, including one in which different resolution data sets for LST, NDVI, and Rainfall were used to detect coincident anomalies associated with disease outbreaks. Anyamba A, Small JL, Britch SC, Tucker CJ, Pak EW, Reynolds CA, et al. (2014) Recent Weather Extremes and Impacts on Agricultural Production and Vector-Borne Disease Outbreak Patterns. PLoS ONE 9(3): e92538.** [**https://doi.org/10.1371/journal.pone.0092538**](https://doi.org/10.1371/journal.pone.0092538)**. Beyond 30m, environmental fluxes, including rainfall and temperature, are comparable, and furthermore, we are dealing with monthly data where most effects of variability are averaged out or minimized (temporal and spatial smoothing).**

--------------------

Results

-Does the analysis presented match the analysis plan?

-Are the results clearly and completely presented?

-Are the figures (Tables, Images) of sufficient quality for clarity?

5. Reviewer #1: Yes

**Response: Thank you!**

6. Reviewer #2: The analysis seems adequate to adress the experimental design. The ststistical tests seem appropriet for the types data presented.

The tables are well preseed and so are the other illustrations.

In table 1, colum 3 data does not add up to 204. It adds up to 194. Please check and correct

**Response: Thank you for this comment. We have updated the table, and it is now correct. The column adds up to 204.**

**Revision: Table 1**

Reviewer #3: The results are clearly and completely presented, very informative details are given in the supplemental. Figures and tables are of very good quality for clarity. Some suggestions should be taken into account:

7. L274: "By modeling such relationships, we hoped to control for ..." In the text, it remains unclear what your results show. The sentence implicit asks for "...., but....". This will follow only later in

**Response: We agree that this is confusing and have clarified it in the text (lines 328-329). Additionally, based on comment #41 below, we have moved this information into the Methods section of the manuscript.**

8. L282. Please rephrase your paragraph introduction.

**Response: We rephrased this paragraph and have focused on the results of extreme climate’s effect on egg abundance (lines 379).**

9. L299: Even if there is a repetition in the wording, I suggest to keep the word "egg abundance" instead of vector abundance.

**Response: Thank you for this comment. We replaced “vector abundance” with “egg abundance” where appropriate throughout the manuscript to allow for greater clarity.**

10. L301ff: Doubling the information about OR in text while it is shown already in table 4 can be questioned.

**Response: Thank you for this comment. It does seem repetitive; however, there is a lot of information in Table 4, which can be very difficult for readers to digest. We believe it is important to highlight significant results in the text so that they are more easily interpretable by readers.**

--------------------

Conclusions

-Are the conclusions supported by the data presented?

-Are the limitations of analysis clearly described?

-Do the authors discuss how these data can be helpful to advance our understanding of the topic under study?

-Is public health relevance addressed?

11. Reviewer #1: No

**Response: Conclusions based on our data analyses can be found in lines 779-849. Limitations are described in lines 904-935. The importance of the data and its public health relevance are includes in lines 877-895 and 936-943.**

12. Reviewer #2: The conclusions are supported by the analysis which is sufficiently rigorous. This being a review paper, the authors should discusd the effects of temperature on dengue virus extrinsic incubation period (EIP).

**Response: Thank you for this comment. We have included impacts of temperature on EIP in the discussion.**

**Lines 868-876: “For example, previous studies of dengue dynamics in China have observed nonlinear statistical relationships between vectors, human incidence, and climate [38-39]. This nonlinear relationship is in part driven by the extrinsic incubation period (EIP), the time it takes for the virus to disseminate in the mosquito, and mosquito lifespan. EIP duration and the mosquito lifespan are temperature-dependent. EIP becomes faster at higher temperatures, but the mosquito must survive longer than the EIP for transmission to occur. The 29° C thermal optima derived from the full suite of Ae. *aegypti* temperature-dependent traits and widespread occurrence of the Ae. *aegpyti* vector throughout Africa, suggests that as temperatures increase, dengue burden is also likely to increase throughout sub-Saharan Africa [41].”**

13. In the case of malaria rainfall has a near linear relationship with vector abundance, within 3-4 months within the rainy season, Threaftet ecological changes disruptt this relationship. However imperical evidence indicates that temperature has an exponential effect on the EIP even when the small temperature changes occurring at a critical transmission threashold may not be statistically significant.The authors have been explicit on the limitations of their study and in particular the use of satellite data. Aquisation is station data can be an expensive undertaking. However this problem can be resolved by collaborating with the Kenya Meorological Department. Normally they operate all weather and climate based early warning systems and are there for key stakeholders in these types of studies.

**Response: Thank you for this comment. Please see response to comment #31 of the Editor below addressing the question of satellite-derived data.**

14. The authors recognize that thier research could lead to the development of an weather based early warning system. It would be usefull to discuss the effects is ENSO and IOD on extreme weather in Kenya and East Africa.

**Response: Thank you for this comment. This is absolutely an important point, and we have included a discussion about ENSO/IOD in our discussion.**

**Lines 886-894: “An improved understanding of the relationship between extreme climate and dengue transmission can also allow for the development of climate-based early warning systems in Kenya. The El Niño Southern Oscillation (ENSO) and Indian Ocean Dipole (IOD) are responsible for changes in the sea-surface temperature that moderate seasonal rainfall and temperature variability in eastern Africa. As such, there is an increased likelihood of extreme climate events during extreme ENSO and positive IOD. By discerning the spatiotemporal relationship between extreme climate and dengue transmission, we can use ENSO and IOD indices to predict periods of heightened arboviral infection risk.”**

15. Reviewer #3: The conclusions are supported by the presented data and the limitations are mainly discussed (L439ff). Please discus possible problems in PCR or Elisa which can occur transporting the blood from Kenia to Standford and which cross reactions may occur? Was capacity building taken into consideration to do the blood measures in Kenia?

**Response: Thank you for these comments. We have clarified in the text that blood samples were tested in both Kenya and at Stanford. Transportation of blood sample shipments from Kenya to Stanford was done on dry ice, and samples were kept in -80° C freezers. Nothing will cross-react on PCR. Flaviviruses can potentially cross-react for the ELISA, but studies have shown that dengue virus (DENV) is most abundant in Kenya. There is some West Nile virus (WNV), there is no yellow fever (people are not routinely vaccinated), and in testing thousands of samples for Zika virus (ZIKAV), we have never found a positive. The Kenyan Ministry of Health even reported a ZIKAV seroprevalence of <0.1% in Kenya. Capacity building was an important part of this work and we provided additional training to local health officials to conduct PCRs and ELISAs in Kenya.**

**Lines 928-935: “Underreporting and misclassification of fever can influence results as well. Since DENV is heavily underreported in the region, all enrolled participants were tested for dengue and malaria. In a study of undifferentiated fever in Kenya between 2014-2017, 150 (51.5%) of 291 participants with dengue viremia were malaria smear positive, suggesting a large overlap in infections [35]. Additionally, flaviviruses, which include West Nile virus (WNV), ZIKA virus (ZIKAV), and yellow fever virus (YFV), have the potential to cross-react with DENV. However, WNV has a comparatively lower seroprevalence compared to DENV [34] while ZIKAV and YFV are essentially absent from our study sites.”**

16. Future research is outlined, and the public health relevance is addressed (L464ff).

L374-384: The terms urbanization and rural areas are only introduced in the discussion. In this paragraph you bring up new results and discus them. I would have expect these additional results which are shown in the supplemental already described at the end of the results chapter "Ae. aegypti eggs".

**Response: We agree that this is confusing. We have clarified this information in the Methods, explicitly describing the four study sites and introducing the term, urbanization, earlier on.**

**Lines 180-182: “This study took place at two sites in western Kenya, Chulaimbo (rural) and Kisumu (urban), and two sites in coastal Kenya, Ukunda (urban) and Msambweni (rural) (Fig. 1). The study sites vary in DENV burden, climate, geography, population size, and urbanization.”**

17. L411: Is an adaptation of Ae. aegypti towards warmer temperatures expected in the literature?

**Response: No, we understand very little about the capacity for *Ae. aegypti* to adapt to changing temperatures.**

18. L422: "...that mosquito abundance and dengue risk do not necessarily share a linear association..". Please compare with further literature from other regions (e.g. Ruiyun 2018 PNAS).

**Response: This is a great point, and we have added this comparison to the literature from other regions.**

**Lines 860-861: “For example, previous studies of dengue dynamics in China have observed nonlinear statistical relationships between vectors, disease incidence, and climate [38-39].”**

19. L432: Does underreporting or misclassification of fever can play a role, too? This is often described in other studies (e.g. WHO 2019, update of the dengue situation).

**Response: Thank you for this comment. Our manuscript now clarifies that we also tested individuals for malaria, the most common cause of febrile illness in individuals in Kenya.**

**Lines 920-924: “Since DENV is heavily underreported in the region, we attempted to unmask such infections by testing all acute febrile cases, including those with malaria. In a study of undifferentiated fever in Kenya between 2014-2017, 150 (51.5%) of 291 participants with dengue viremia were malaria smear positive, suggesting a large overlap in infections.”**

20. L432ff: Besides indirect effects of temperature on mosquito abundance, direct effects of temperature on the transmission should be mentioned here, too (e.g. temperature depend extrinsic incubation period of dengue, reviewed in Tjaden 2013 PlosNTD).

**Response: Thank you for this comment. We have included impacts of temperature on EIP in the Discussion.**

**Lines 861-868: “This nonlinear relationship is in part driven by the extrinsic incubation period (EIP), the time it takes for the virus to disseminate in the mosquito, and mosquito lifespan. EIP duration and the mosquito lifespan are temperature-dependent. EIP becomes faster at higher temperatures, but the mosquito must survive longer than the EIP for transmission to occur [37]. The 29° C thermal optima derived from the full suite of Ae. aegypti temperature-dependent traits and widespread occurrence of the Ae. aegpyti vector throughout Africa, suggests that as temperatures increase, dengue burden is also likely to increase throughout sub-Saharan Africa [41].”**

--------------------

Editorial and Data Presentation Modifications?

Use this section for editorial suggestions as well as relatively minor modifications of existing data that would enhance clarity. If the only modifications needed are minor and/or editorial, you may wish to recommend “Minor Revision” or “Accept”.

Reviewer #1: (No Response)

21. Reviewer #2: Please check additions in table 1 column 3.

**Revision: Thank you for this comment. We have updated the table, and it is now correct. The column adds up to 204.**

22. Reviewer #3: L107: "... higher monthly rainfall up to 16.2mm over the past decade." The absolute value of mm increase within decades, does not help to understand if this is an (nearly) extrem value or not. Maybe you add the percentage of the increase.

**Response: It is true that this does not provide us with an understanding of whether the rainfall value is extreme or not. It was included to shed light on previous findings which support a level at which more extreme values might limit dengue transmission. Unfortunately, the reference does not provide information regarding the percentage of the increase or deviation from the monthly mean, which would be useful to include.**

23. L151: "0.05° MOD11C3 " you may add the km as done for rainfall "gridded at 0.1° x 0.1° (≈11 km)".

**Response: We have added the km as done for rainfall.**

**Lines 194-195: “LST data are available at a spatial resolution 0.05° x 0.05° (≈5.5 km), thus allowing us to extract monthly LST for a 30 km x 30 km grid centered on each study location.”**

24. L224: Please change Aedes aegypti in Ae. aegypti.

**Revision: This has been changed to *Ae. aegypti*, as requested (line 297).**

25. L258: "There is no standard for such designations, ", you can justify your approach by citing IPCC 2012.

**Response: Thank you for this comment! We justified our approach in the text by citing IPCC 2012 (lines 353-354).**

26. L261: Table 1. Distribution of Rainfall Categorization: you may use different background color or grey to divide between sites prone to drought events (Chulaimbo, Kisumu) and those sites prone to flood events (Msambweni, Ukunda). To better understand this result, it would be helpful to support the reader with a map of the elevation of Kenia, e.g. within the study site map.

**Response: Thank you for this comment; we agree that this would be very helpful for readers. We have updated our study site map to illustrate the elevation of Kenya and have used different background colors to differentiate the western sites from the coastal sites.**

**Revision: Table 1, Table 2, and Figure 1.**

27. L344: Change table 5 to table 4.

**Response: Thank you; this has been revised in the text.**

28. L395: Do you mean "transmission by Ae. aeypti..." or "transmission for dengue"?

**Response: Thank you; this paragraph has been altogether rewritten.**

--------------------

Summary and General Comments

Use this section to provide overall comments, discuss strengths/weaknesses of the study, novelty, significance, general execution and scholarship. You may also include additional comments for the author, including concerns about dual publication, research ethics, or publication ethics. If requesting major revision, please articulate the new experiments that are needed.

Reviewer #1: (No Response)

29. Reviewer #2: Climate change is having and impact on the geographic range, seasonalty and the evolition of vector-borne diseases (VBD) epidemics. Among the adaptation options is the ability to predict these trends and to develop early warning systems to enable pre-emptive interventions. Extreme weathet events are often associated with VBD epidemics. It is criticslly important to understanf the biology of trandmission in relationship to the extremr weathet events. This paper highlights efforts towards that direction. As an exploratory study It has indicated how available entomological, virological and meteorological data analysis may lead to the understanding of extreme weather events on dengue fever.

The weakness of the paper lies in the failure to include critical and well known temperature threasholds of dengue virus transmission biology. Equaly rainfal has lower and upper threasholds that either support vector breeding or disrupt it. Thus statisticall defined extremes must take cognisance of biologically relevant thresholds.

Although the paper has discussed the stelite data limitations it shout highlight these other limitation so that they are avoided in futurr work.

The research has no outstanding ethical issues.

The paper should address these concerns without undertaking new data analysis.

**Response: Thank you for the very helpful comments! There is still a lot unknown about whether these thresholds, measured in controlled laboratory settings for individual traits, actually lead to defined thresholds in reality. As such, it would be valuable to identify statistically defied extremes based on field data to contextualize our results with laboratory findings of temperature thresholds. In the laboratory-setting, there are previously identified temperature thresholds for dengue virus transmission by *Ae. aegypti*, which we have discussed extensively in the Introduction and Discussion (Mordecai et al., 2017). Relationships between such thresholds and dengue transmission are not as clearly delineated for rainfall. This is an important point which should be considered for other studies.**

**Lines 123-148: “As such, the relationship between rainfall and dengue transmission is not clearly delineated…However, much remains unknown about whether such laboratory-measured temperature thresholds lead to defined thresholds in reality… We hope to better contextualize previous temperature thresholds and trends associated with DENV transmission using field data in Kenya.”**

30. Reviewer #3: This study is topically and of high interest. The authors aim to close a knowledge gap on the effects on extreme events on mosquito-borne diseases. The results can be helpful to consider mosquito control actions and public health interventions in time.

in my opinion a short introduction in the wording with regard to climate change is missing, including trends, climate variability and extreme events (e.g. extreme events following natural internal climate variability (which is the element of climate variability that arises spontaneously within the climate system even in the absence of forcings, see IPCC AR5 2014) or due to climate warming). Also it would be helpful, if you can refine your wording with regard to "extreme weather events" and "extreme climate" (see IPCC 2012, Managing the risk of extreme events, 3.1.2.).

**Response: Thank you for this comment. We have included a description of how climate variability can influence extreme climate events in the introduction and consistently use “extreme climate events” throughout the remainder of the manuscript.**

**Lines 82-85: “Extreme climate events result from both natural internal climate variability and climate warming [40]. Climate variability arises spontaneously within the climate system even in the absence of climate forcings [40]. With regards to climate warming, sea-surface temperature increases of 1-2° Celsius can result in greater trapped greenhouse gas molecules and energy flux in the lower atmosphere, resulting in stronger, more unpredictable weather patterns [1].”**

--------------------

The paper presents results of the effect of land surface temperature, rainfall and season on mosquito vectors of dengue fever virus and infection patterns in children in four sites in Kenya – Ukunda, Masambweni, Chulaimbo and Kisumu. Data were collected between 2013 and 2019 and temperature and rainfall anomalies derived for the periods 2000 – 2019 and 1983 to 2019, respectively, and used as predictors. Statistically significant results were obtained for mosquito populations but not with infection rates.

Major comments

The paper however has a number of weaknesses that renders it unsuitable for this journal.

31. The authors have used satellite climate (LST and rainfall) data with no comparison, reference to ground measurements from meteorological stations in the study areas. Satellite data have lower resolution and always averaged over vast regions. It often makes a lot of sense to use such data for analyses conducted over wide geographical areas and not for areas that are too close to present meaningful comparisons. In fact anomalies for rainfall data are estimated at the county level and assigned to study sites. This effectively reduces the power of the study and it might have contributed to the lack of effect of predictors on infection patterns in children.

**Response: We agree with the Reviewer that the satellite data is more coarse than ground measurements from meteorological stations and could affect comparisons between paired coastal and western sites that are geographically close together; however, the benefit of using satellite-derived climate data is the ability to compare deviations in recent climate conditions from long-term records, which can only be obtained through satellite data. Given the focus of our study was to understand extreme climate conditions, we believe using satellite data, that limits our ability to make spatial comparisons, is useful in allowing us to make temporal comparisons. We have added a sentence to the Discussion about this topic. We would also like to highlight that the satellite-derived climate data for both LST and rainfall are validated products that have been used for over 20 years. The accuracy of daily MODIS LST product at 1-km resolution was validated in 11 clear-sky cases with in situ measurement data collected in field campaigns in 2000 and 2001 and has an estimated accuracy of +/-1°C [31]. The rainfall product is derived from a combination of satellite data, models, and rain gauge measurements; therefore, it includes ground measurements from across Africa. Additionally, we see strong correlations between LST and temperature between 2013-2019 across all four sites, as seen below.**

**Lines 897-900: “Due to the lack of long-term climate data available for analysis, this study makes use of satellite-derived data for four study sites in Kenya, which can provide us with a reference for the effect of extreme climate events more generally in the region. As such, satellite-derived climate data offers the ability to compare deviations in recent climate conditions from long-term records.”**

32. No data are provided to show the ages of children used, their distribution by households etc to gauge whether their infection patterns provided a good representation of the study areas used. If young children were used, then it can be argued that their force of infection was too low to be of practical significance given that they are raised in doors most of the time. Published data show that Aedes mosquitoes are day biters. They are more likely to transmit the disease during the day than the night like other mosquitoes such as Anopheles which transmits malaria. Older people are expected to come into contact with mosquitoes as they work outside.

**Response: We very much appreciate this comment. Unlike most developed nations, children in Kenya spend significant time outside. “Baby school” starts at 3, and many children attending baby school actually walk to school and play outside. We have provided additional information regarding the range of ages for children included in the study.**

**Lines 266-277: “Dengue (DENV) incidence was assessed based on blood samples collected from children with acute febrile illness presenting to one of the four study sites (Mbaka Oromo Health Centre in Chulaimbo, Obama Children’s Hospital in Kisumu, Msambweni District Hospital in Msambweni, and Ukunda/Diani Health Center in Ukunda) (Fig. 1). The study population consisted of 7,653 children less than 18 years of age (median = 5 years [1 year, 15 years]). Unlike other places around the world, children in Kenya spend a lot of time outside during the day, which is when Ae. aegypti are actively biting. Blood samples were tested on site in Kenya by Ministry of Health collaborators at Msambweni District Hospital for coastal Kenya sites and KEMRI Kisian Field Station for western Kenya sites; samples were also tested at Stanford University.”**

Minor comments

33. Line 163 – 165: Reference periods for the estimation for anomalies for LST and rainfall differ. This is confusing, it might be better to use a common reference time period for both.

**Response: The reference periods for the estimation for anomalies for LST and rainfall differ simply because of data availability. As referenced in the WMO’s Guidelines on the Calculation of Climate Normals, the inclusion of all available observations allows for a more accurate characterization of the study locations’ climate, especially when fewer than 30 years of data are available [26].**

**Lines 210-213: “As referenced in the WMO’s Guidelines on the Calculation of Climate Normals, the inclusion of all available observations allows for a more accurate characterization of the study locations’ climate, especially when fewer than 30 years of data are available [26]; as a result, the reference periods for LST and rainfall differ for this study.”**

34. Line 177 – Flood is categorised as a positive rainfall deviation above 90% threshold. His categorization is done without considering the topography of the study areas not the length of time this this anomaly has been observed. A positive anomaly for one month is not enough to cause floods in some places. The interpretation of the effect of this variable therefore is not justifiable. This is why in Line 291, it is observed that flooding increases egg load. This is not usually true especially if flushing was to occur

**Response: We have defined a flood as an abnormally wet period in which there is a rainfall anomaly above the 90^th^ percentile. This definition is specific to this study and is defined in lines 235-236. Kenya has undergone tumultuous changes in climate patterns over the past five years, and as such, this study is interested in such anomalies.**

35. Line 185: Drought is defined as a dip in rainfall levels by at least 10%. However, drought usually occurs where there has been prolonged period of dry weather for several months. This classification, as that identified above, brings confusion and makes data interpretation difficult to follow

**Response: We have defined a drought as an abnormally dry period in which there is a rainfall anomaly above the below the 10th percentile. This definition is specific to this study and is defined in lines 236-237.**

36. Line 216 Replace “is” with “was”

**Revision: This has been revised in the manuscript.**

37. Line 225 Indicate whether the ordinal logistic regression model used satisfied the proportional odds assumption

**Response: Thank you very much for raising this concern. Two of the ordinal logistic regression models (BG-traps and Prokopack) did not satisfy the proportional odds assumptions based on a Brant Test. Based on this comment and the comment below, we used generalized log-linear mixed models instead. The data satisfies all the assumptions for generalized log-linear mixed models (lines 321-329).**

38. Line 227 Mosquito counts are not expected to follow a normal distribution. Many studies often transform the raw data e.g. using log transformation

**Response: Thank you for this comment. We have replaced our ordinal logistic regression with a generalized log-linear model in which we have transformed the data. Additionally, we have acknowledged the non-normality of the data (lines 321-329).**

39. Line 245 what is the justification for using 7 as the cut off for the number of cases

**Response: 7+ cases were defined as “high” DENV transmission because such a threshold characterized the 90+ percentile for dengue cases. Monthly DENV infections followed a non-normal distribution, and as such, we defined the upper 10% of monthly cases as “high.”**

**lines 333-335: “7+ confirmed DENV infections represent the upper 10% of monthly cases and can thus be defined as “higher” than normal in a region with low levels of DENV transmission.”**

40. Line 257 What are the groups being considered?

**Response: “Group” refers to the individual climate event designations, whether that is flood, drought, normal rainfall, cold wave, heat wave, or normal LST.**

**lines 351-354: “Establishing defined thresholds for our categorization of extreme climate events allows us to compare related climatic variables of interest between types of extreme climate.”**

41. Line 274 - 276 fits well in the methodology. It is trying to justify the type of analysis done

**Response: Thank you for this comment, and we agree. This information has been moved to the Methods.**

42. Line 376 Urbanization not introduced in the methodology

**Response: We agree that this is confusing. We have clarified this information earlier on in the methodology and more explicitly described the four study sites.**

**Lines 180-182: This study took place at two sites in western Kenya, Chulaimbo (rural) and Kisumu (urban), and two sites in coastal Kenya, Ukunda (urban) and Msambweni (rural) (Fig. 1). The inclusion of several different study sites allows us to consider the effects that site-specific effects can have on DENV transmission, including geography and urbanization, which considers human population density.”**

Line 392 How can heat wave affect Chulaimbo alone, and not together with the other site in the same county? Are there other variables that were not captured

**Response: Thank you for this comment. It is entirely possible to have very localized anomalies not affecting other areas. This can be due to local topographic effects, wind, etc., all of which are important to consider and relevant for climate observations.**

**Lines 806-807: “Even sites that are close in proximity to one another can experience different climate anomalies due to differences in local topography, wind, etc.”**
